# Visualizing Central Vessels of Hepatic Angiomyolipoma Devoid of Fat Using a 2D Multi-Breath-Hold Susceptibility-Weighted Imaging

**DOI:** 10.1155/2015/197431

**Published:** 2015-05-31

**Authors:** Ruo-Kun Li, Meng-Su Zeng, Jin-Wei Qiang

**Affiliations:** ^1^Department of Radiology, Jinshan Hospital, Fudan University, 1508 Longhang Road, Shanghai 201508, China; ^2^Department of Diagnostic Radiology, Zhongshan Hospital, Fudan University, 180 Fenglin Road, Shanghai 200032, China

## Abstract

Epithelioid hepatic angiomyolipoma (Epi-HAML) is a rare benign mesenchymal tumor with malignant potential. Most of Epi-HAML contains no or only a minimal amount of adipose tissue and poses a diagnostic challenge. Central vessels are characteristic imaging finding of Epi-HAML, which usually were displayed by dynamic contrast imaging. In this paper, we displayed the central vessels of Epi-HAML invisible on conventional MR images using a new developed abdominal susceptibility-weighted imaging (SWI). To the best of our knowledge, this is the first description for the role of SWI in characterization of Epi-HAML.

## 1. Introduction

Hepatic angiomyolipoma (HAML) is a rare benign mesenchymal tumor, which is composed of variable amounts of fat tissue, smooth muscle cells, and vessels at histopathology [1, 2]. Depending on the dominant type of the smooth muscle cell, HAML can be subcategorized into epithelioid, spindle, and intermediate forms [3]. Epithelioid hepatic angiomyolipoma (Epi-HAML) is usually devoid of adipose tissue and preoperative diagnosis is quite difficult. A few studies indicated central vessels as characteristic imaging findings [4, 5]. Dynamic contrast imaging could detect large vessels within the tumor. However, microvessels are difficult to visualize due to the partial volume effect.

Susceptibility-weighted imaging (SWI) is an emerging MRI technique. In neuroimaging, SWI is a 3D, fully velocity-compensated, gradient echo sequence. Brain SWI can be used to visualize smaller intracerebral veins and other sources of susceptibility effects, such as hemosiderin, ferritin, and calcium [6, 7]. We have developed a new 2D abdominal SWI sequence for liver imaging at a 3T MR scanner. Our previous studies showed that abdominal SWI technique could improve the detection of siderotic nodules in cirrhotic patients and intratumoral hemorrhages within hepatocellular carcinoma [8, 9]. In this paper, we report a case of Epi-HAML devoid of adipose tissue. The abdominal SWI could well visualize intratumoral microvessels invisible on conventional MR images. To the best of our knowledge, there is no description for the role of SWI in characterization of Epi-HAML.

## 2. Case Report

A 27-year-old woman complained of upper abdominal discomfort for 2 weeks without fever, jaundice, or weight loss. She denied any history of exposure to specific carcinogens. A review of family history was unremarkable. Physical examination revealed hepatomegaly. Liver function tests were normal. Serology was negative for hepatitis B and hepatitis C. Levels of tumor markers alpha-fetoprotein (AFP), carcinoembryonic antigen (CEA), carbohydrate antigen 19-9 (CA 19-9) were normal.

**Figure 1:**
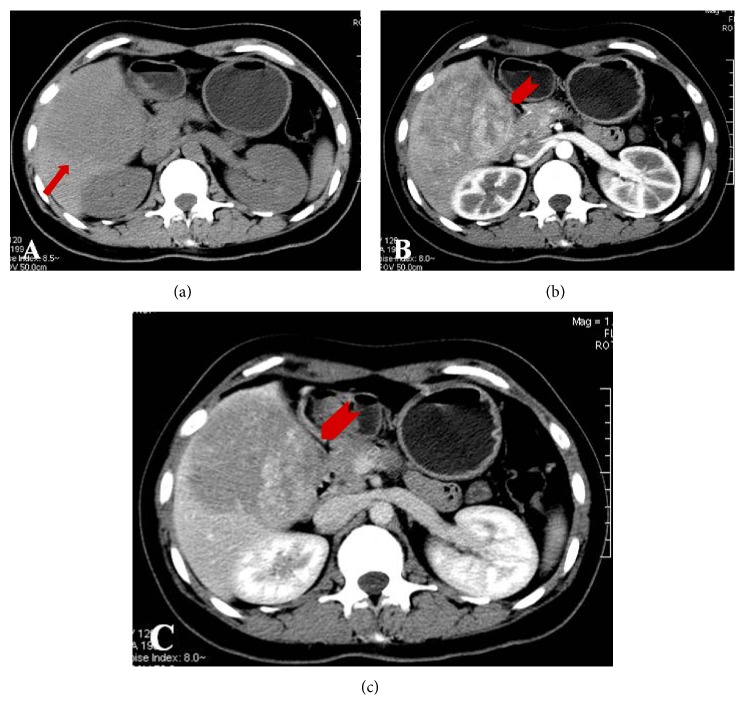
Unenhancement CT (a) images reveals a large well-demarcated hypoattenuate mass in the right lobe of the liver (arrow). No fat tissue is detected at CT images. The mass shows heterogeneously intense enhancement at hepatic arterial phase ((b) arrowhead) and persistent enhancement at portal venous phase ((c) arrowhead).

Due to the nonspecific complaints, a biphasic contrast-enhanced CT scanning of the upper abdomen was performed using GE 64-detector CT scanner with hepatic arterial and portal venous phase imaging with delays of 30 s and 70 s. CT images revealed a large well-demarcated hypoattenuate mass with heterogeneously intense enhancement on dynamic contrast imaging in the right hepatic lobe. No fat components were detected at CT images (Figure 1). Subsequently, she underwent MR examination at a 3T MR scanner (Magnetom Verio; Siemens, Erlangen Germany) using a 12-channel body coil. Conventional imaging sequences included respiratory-navigated T2-weighted turbo spin echo sequence, breath-hold T1-weighted fast low-angle shot sequence, and 3D T1-weighted fat-suppressed spoiled-echo sequence which was performed for dynamic imaging once before and three times after bolus injection of 20 mL gadopentetate dimeglumine. A new abdominal 2D SWI sequence was performed precontrast using a multi-breath-hold gradient echo sequence with the following parameters: repetition time, 150 ms; echo time, 10 ms; section thickness, 5 mm; intersection gap, 1 mm; matrix, 187 × 384; voxel size 1.5 × 1.0 × 0.5 mm^3^, flip angle, 20°; bandwidth, 189 Hz/pixel; field of view 285 × 380 mm. Parallel imaging was performed using the generalized autocalibrating partially parallel acquisition (GRAPPPA) with an acceleration factor of 2. The total acquisition time was 49 seconds, including 3 times of breath-hold with 10 seconds of free breathing. SWI postprocessing was performed according to [9].

**Figure 2:**
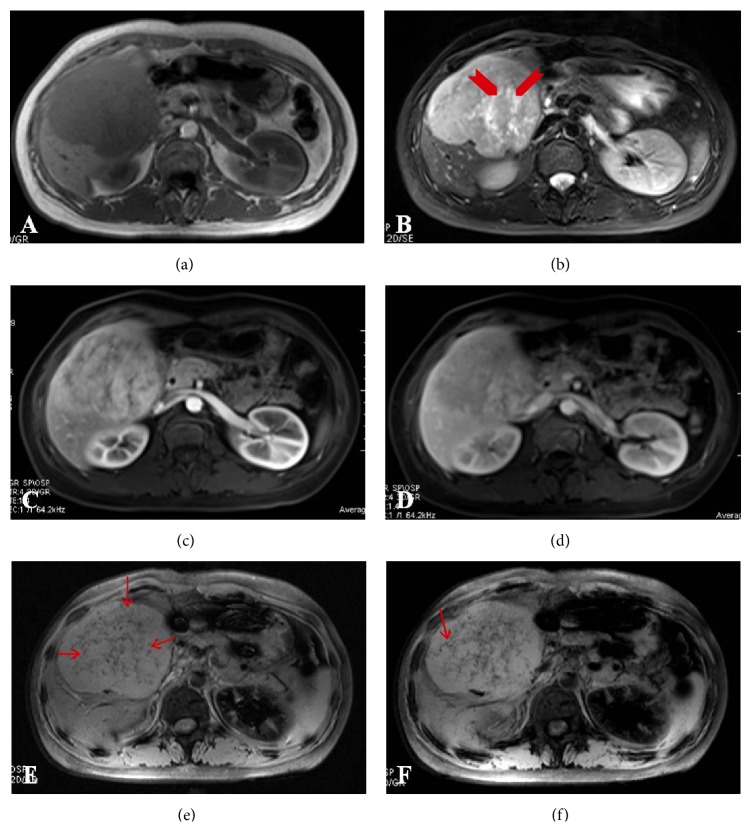
The mass shows inhomogeneous hypointensity on T1WI (a) and hyperintensity on T2WI ((b) arrowhead) with inhomogeneously intense enhancement at arterial phase (c) and persistent enhancement at portal venous phase (d). SWI images (e) reveal numerous branching punctate or curved central microvessels (arrow). The continuity of the central microvessels could be better visualized on MinIP images of SWI ((f) arrow).

At MR images, the mass showed heterogeneous hyperintensity on T1WI (Figure 2(a)) and clearly hyperintensity on T2WI (Figure 2(b)). No fat components were detected at MR images. Dynamic imaging showed a heterogeneous hypervascular mass after gadolinium administration (Figures 2(c) and 2(d)). SWI could visualize central microvasculature with better conspicuity (Figure 2(e)), which appeared as branching curvilinear configuration with hypointensity on minIP images of SWI (Figure 2(f)).

According to the conventional MR images and clinical features of this patient, a benign hypervascular tumor of the liver, for example, focal nodular hyperplasia, adenoma, or HAML, was suspected. However, when the SWI findings were referred, the diagnosis of HAML was considered.

**Figure 3:**
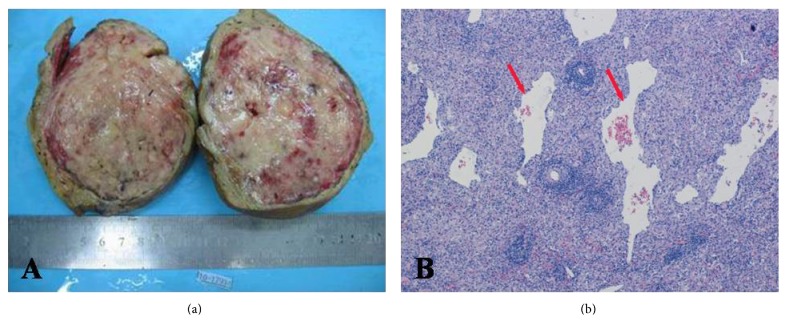
Gross specimen (a) shows the tumor is yellowish mixed with white colored tissue and red vessels without hemorrhage or necrosis. Microscopy views ((b) hematoxylin and eosin stain, original magnification ×20) showed that the tumor is composed of epithelioid cells and malformed vessels (arrow).

The patient underwent surgery for tumor resection with informed consent. The cut surface of the tumor was yellowish mixed with white colored tissue and red vessels. The mass was well defined, but no fibrous capsule was observed (Figure 3(a)). Histopathologically, there were large vessels and many small thin-walled vessels surrounded by proliferated spindle-shaped tumor cells, which showed strongly positive for a melanocytic cell-specific mononuclear antibody (HMB-45) on immunohistochemical stain and muscle-specific actin stain (Figure 3(b)). The tumor was completely devoid of fat at histopathological examination. Pathological diagnosis of Epi-HAML was confirmed.

## 3. Discussion

Hepatic angiomyolipoma (HAML) is an unusual mesenchymal neoplasm composed of blood vessels, smooth muscle, and adipose cells [1, 2]. Epi-HAML is a rare subtype of HAML which is prone to occur in females without specific symptoms. At present, it is becoming increasingly clear that Epi-HAML should be regarded as tumors of uncertain malignant potential. Rare cases of Epi-HAML with tumor recurrence, distant metastasis, and vascular invasion were reported. Surgical resection and carefully follow-up are recommended in clinical practice [10–12].

The radiological appearance of HAML varies widely due to the fact that the distribution and relative proportion of three components varies widely. The fat content of typical HAML produces a characteristic appearance on imaging studies that can be easily diagnosed by CT or MRI [13, 14]. However, most of Epi-HAML contains no or only a minimal amount of adipose tissue and poses a diagnostic challenge radiologically. In the present case, CT and MRI either did not detect any adipose component, consistent with previous reports.

Central punction or filiform vessels was considered as a characteristic imaging feature of Epi-HAML compared with other hypervascular hepatic tumors [4, 5]. Large vessels within the tumor could be visualized by dynamic contrast imaging after administration of contrast media. However, microvessels are usually invisible due to partial volume effect and limited spatial resolution. In addition, for patients with risk of allergic reaction and renal insufficiency, dynamic contrast imaging could not be performed. In the present case, no definite vessels were detected on CT and conventional MRI images. However, SWI visualize central microvasculature with better conspicuity, which appeared as curvilinear configuration with hypointensity. MinIP images of SWI could visualize continuity of microvessel, which is different from intratumoral vessels within HCC, which is immature and discontinuity in morphology.

SWI has been applied to visualize intracerebral venous structures using deoxy-Hb as intrinsic contrast agent in neuroimaging. The magnetic susceptibility of deoxy-Hb causes an average intravascular proton frequency shift and leads to a phase difference between venous blood and the surrounding tissue. To enhance the visibility of the venous structures, the magnitude images were manipulated with the phase data by the use of the phase mask to maximize the negative intensities of the veins. The unique phase mask technique renders SWI exquisitely sensitive to small susceptibility changes. Consequently, SWI was sensitive enough to susceptibility changes to visualize intracerebral venous anatomy at submillimeter resolution. In this case, numerous microvessels could be better visualized on SWI images compared with the conventional MR images [15, 16].

Epi-HAML should be carefully differentiated with other hypervascular hepatic lesions, such as hepatocellular carcinoma (HCC) and focal nodular hyperplasia (FNH). The presence of arterial hypervascularity and venous/equilibrium washout is generally considered to be specific enhancement pattern for the diagnosis of HCC on dynamic imaging. Additionally, capsule could be found in most of HCCs but not seen in Epi-HAML. Clinical history of chronic liver disease and evaluated AFP level also suggest the diagnosis of HCC. FNH typically shows brisk homogeneous enhancement on the arterial phase except the central scar which shows hyperintensity on T2WI and delayed filling in [4, 5, 17].

Our study has a limitation as a case report. We only previously described the fact that SWI was superior to conventional MRI sequences for visualizing microvessels in only one Epi-HAML patient. Hence, it is necessary to determine its statistical significance with large clinical series in further studies.

In summary, our case report shows that SWI may be a potential too for visualizing central microvessels of Epi-HAMs, which may be useful for making correct preoperative diagnosis.
